# Ability of parasternal intercostal muscle thickening fraction to predict reintubation in surgical patients with sepsis

**DOI:** 10.1186/s12871-024-02666-8

**Published:** 2024-08-22

**Authors:** Mina Adolf Helmy, Ahmed Hasanin, Lydia Magdy Milad, Maha Mostafa, Walid I Hamimy, Rimon S Muhareb, Heba Raafat

**Affiliations:** Department of Anesthesia and Critical Care Medicine, Faculty of Medicine, Cairo University, Cairo, Egypt

**Keywords:** Parasternal thickening, Diaphragmatic excursion, Mechanical ventilation, Spontaneous breathing trial, Failed weaning, Reintubation

## Abstract

**Objectives:**

We aimed to evaluate the ability of the parasternal intercostal (PIC) thickening fraction during spontaneous breathing trial (SBT) to predict the need for reintubation within 48 h after extubation in surgical patients with sepsis.

**Methods:**

This prospective observational study included adult patients with sepsis who were mechanically ventilated and indicated for SBT. Ultrasound measurements of the PIC thickening fraction and diaphragmatic excursion (DE) were recorded 15 min after the start of the SBT. After extubation, the patients were followed up for 48 h for the need for reintubation. The study outcomes were the ability of the PIC thickening fraction (primary outcome) and DE to predict reintubation within 48 h of extubation using area under receiver characteristic curve (AUC) analysis. The accuracy of the model including the findings of right PIC thickening fraction and right DE was also assessed using the current study cut-off values. Multivariate analysis was performed to identify independent risk factors for reintubation.

**Results:**

We analyzed data from 49 patients who underwent successful SBT, and 10/49 (20%) required reintubation. The AUCs (95% confidence interval [CI]) for the ability of right and left side PIC thickening fraction to predict reintubation were 0.97 (0.88–1.00) and 0.96 (0.86–1.00), respectively; at a cutoff value of 6.5–8.3%, the PIC thickening fraction had a negative predictive value of 100%. The AUCs for the PIC thickening fraction and DE were comparable; and both measures were independent risk factors for reintubation. The AUC (95% CI) of the model including the right PIC thickening fraction > 6.5% and right DE ≤ 18 mm to predict reintubation was 0.99 (0.92–1.00), with a positive predictive value of 100% when both sonographic findings are positive and negative predictive value of 100% when both sonographic findings are negative.

**Conclusions:**

Among surgical patients with sepsis, PIC thickening fraction evaluated during the SBT is an independent risk factor for reintubation. The PIC thickening fraction has an excellent predictive value for reintubation. A PIC thickening fraction of ≤ 6.5–8.3% can exclude reintubation, with a negative predictive value of 100%. Furthermore, a combination of high PIC and low DE can also indicate a high risk of reintubation. However, larger studies that include different populations are required to replicate our findings and validate the cutoff values.

**Supplementary Information:**

The online version contains supplementary material available at 10.1186/s12871-024-02666-8.

## Introduction

Nearly one-third of critically ill patients require mechanical ventilation [1]. Prolonged mechanical ventilation is associated with poor patient outcomes and increases the burden on healthcare systems, particularly in the post pandemic era [2]. Conversely, premature extubation usually results in reintubation, which is an independent risk factor for mortality [3, 4].

The optimal point for discontinuing invasive mechanical ventilation should include parameters that prevent both premature extubation and unnecessary prolonged ventilation. The most widely agreed method to reach this point is assessment of the ability of the patient to breathe with no or minimal respiratory support for 30–120 min (spontaneous breathing trial [SBT]) [5]. The success of SBT is usually evaluated on the basis of major respiratory and cardiovascular signs, such as respiratory rate, gas exchange, and hemodynamic parameters. However, there is an increased interest in the evaluation of respiratory muscular dysfunction as an important and unreplaceable measure of weaning eligibility [6]. The evaluation of diaphragmatic function has gained the highest attention for its feasibility at the bedside using point-of-care ultrasound [6]. More recently, the parasternal intercostal (PIC) muscle thickening fraction showed good performance in the evaluation of patients receiving mechanical ventilation during weaning [7, 8]. Evaluation of the PIC has some advantages for being close to the skin and not affected by gaseous distension; these characteristics increased the interest in its use for respiratory muscle assessment because it is much easier than diaphragmatic examination with nearly the same accuracy [7, 8].

The PIC thickening fraction showed good performance in determining SBT failure [7, 9]. However, there are two types of failed SBT: failure to complete the SBT and failure of extubation (reintubation). Previous studies have evaluated the accuracy of PIC in detecting failed SBT, but no studies have evaluated its accuracy for reintubation, which is more serious than failed SBT; thus, detecting reintubation warrants separate investigation. Furthermore, the PIC thickening fraction was not previously evaluated in patients with sepsis. Sepsis can produce several forms of neuromyopathy in different muscles, including the diaphragm [10, 11]; reduces the blood supply of respiratory muscles; and damages contractile proteins [11, 12], aggravating the effect of mechanical ventilation on respiratory muscles [13, 14]. The preferential effect of sepsis on diaphragmatic function indicates that this group of patients requires a separate evaluation of respiratory muscles compared with other critically ill patients [12].

This study aimed to evaluate the ability of the PIC thickening fraction to predict reintubation in surgical patients with sepsis. The secondary aim was to compare the PIC thickening fraction with the diaphragmatic excursion (DE) as predictors of weaning outcomes.

### Patients and methods

This prospective observational study was conducted in the surgical intensive care unit at Cairo University Hospital between September 2022 and July 2023 after institutional research ethics committee approval (Cairo University’s Research Ethics Committee approval no. MD-167-2022). Written informed consent was obtained from the patient’s next of kin.

We consecutively included adult (> 18 years) surgical patients with sepsis who were mechanically ventilated for ≥ 24 h. The diagnosis and management of the patients were based on the latest guidelines [15].

Patients with diaphragmatic paralysis and neuromuscular diseases and pregnant women were excluded.

The attending intensivist assessed the patients for weaning eligibility, which included PaO_2_ > 60 mmHg at positive end expiratory pressure ≤ 8 cmH_2_O, appropriate pH and PaCO_2_, and hemodynamic stability (or on low-dose vasopressors). All included patients were fully conscious and had a good cough reflex [16].

Eligible patients underwent SBT for 120 min using a positive end expiratory pressure of 5 cm H_2_O and pressure support of 5 cmH_2_O. The SBT was considered a failure when any of the following was present: respiratory rate > 35 breaths per minute, increased work of breathing, SpO_2_ < 90% or PaO_2_ < 60 mmHg on FiO_2_ > 0.4, or hemodynamic instability (hear rate > 140 beats per minute or > 20% change from baseline and systolic blood pressure > 180 mmHg or > 20% change from baseline) [16]. Patients who successfully passed the SBT were extubated and were observed for the next 48 h for reintubation. A simple oxygen mask was used to maintain SpO_2_ > 92%. Failed weaning was defined as either failed SBT or the need for reintubation within 48 h after extubation.

An experienced intensivist (LM) who had conducted > 150 previous similar examinations performed ultrasound measurements. Ultrasound examinations were performed using a Versana Essential device (GE Medical Systems Co., Ltd., China) 15 min after initiating the SBT in the semi-sitting position.

### Ultrasound assessment of PIC thickening fraction

**Fig. 1:**
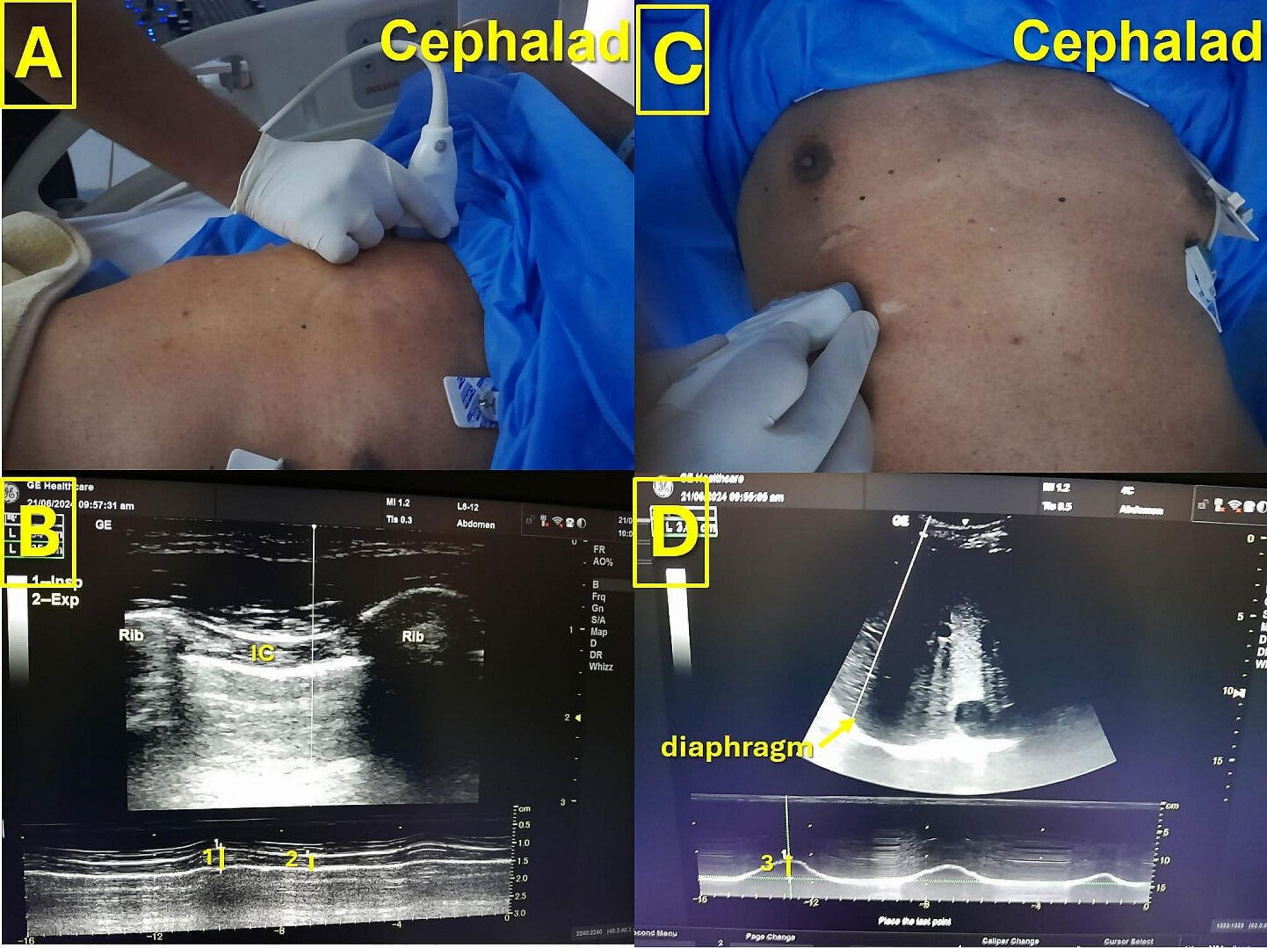
Ultrasound assessment of respiratory muscle. **A**: Transducer placement for assessment of parasternal intercostal thickening fraction. **B**: Inspiratory (yellow line 1) and expiratory (yellow line 2) intercoastal muscle thickness were measured using the M-mode. **C**: Transducer placement for assessment of diaphragmatic excursion. **D**: The amplitude of diaphragmatic muscle (yellow arrow) excursion between inspiration and expiation (yellow line 3) was then measured using the M-mode. IC: intercostal muscle

A high-frequency linear transducer (L6-12-RS, 4–16 MHz) was placed vertically at the second intercostal space, 3 cm lateral to the sternal border. Inspiratory and expiratory muscle thickness was measured using the M-mode. The PIC thickening fraction was calculated as (inspiratory muscle thickness − expiratory muscle thickness/expiratory muscle thickness) ×100% (Fig. 1).

### Ultrasound assessment of DE

A low-frequency curved transducer (4 C-RS, 2–5 MHz) was placed horizontally at one of the lower intercostal spaces on the right anterior axillary line and left midaxillary line for the right and left sides, respectively. The patient was then asked to take a maximum breath, after which the amplitude of excursion between inspiration and expiration was measured using the M-mode (Fig. 1).

The mean PIC thickening fraction and DE were calculated as the average of the right and left side measurements.

Clinicians in charge of patient care were blinded to ultrasound measurements.

The primary outcome was the ability of the PIC thickening fraction to predict reintubation after successful SBT. The secondary outcomes included the accuracy of PIC thickening fraction and DE in predicting successful weaning. The cutoff values obtained from the area under the curve (AUC) analysis for the right PIC thickening fraction and right DE were used to categorize the patients into either positive or negative risk for reintubation. Positive risk for reintubation was considered when the PIC thickening fraction was higher than the obtained cutoff value and/or DE was lower than the cutoff value, and vice versa for negative risk for reintubation. Each patient had either two positive sonographic findings, one positive sonographic finding, or two negative sonographic findings, and the accuracy of this model was assessed. Patient demographic data, comorbidities, Acute Physiology and Chronic Health Evaluation (APACHE) II score, hemodynamic and respiratory parameters (PaO_2_/FiO_2_ ratio PaCO_2_, respiratory rate, and rapid shallow breathing index [RSBI]), cause of mechanical ventilation, days of mechanical ventilation, and source of sepsis were recorded.

### Sample size

Sample size calculation was performed using MedCalc version 18 (MedCalc Software bvba, Ostend, Belgium). Assuming an incidence of reintubation of 20%, a minimum sample size of 45 patients (at least nine reintubation cases) was needed to detect an AUC of 0.8 with the null hypothesis set at 0.5 for a study power of 80% and alpha error of 0.05.

### Statistical analysis

Patients were divided according to 1- weaning outcome (failed weaning, including reintubation or failed SBT versus successful weaning), 2- the need to reintubation versus successful extubation. The Schapiro–Wilk test was implemented to evaluate data distribution; normally distributed data are reported as mean ± standard deviation and were analyzed using the independent sample t-test, whereas skewed data are reported as median (quartiles) and were analyzed using the Mann-Whitney test. Categorical variables are summarized as counts and percentages and were analyzed using the Chi-squared or Fisher’s exact test as appropriate. The AUC was calculated to assess the ability of PIC and DE to predict weaning outcome and reintubation. Youden’s index was used to determine the best cutoff value. Logistic regression was used to calculate odds ratios and 95% confidence intervals (CIs) for the prediction of failed weaning and reintubation. Multivariate analysis using forward selection method was performed to identify independent predictors of failed weaning and reintubation. The model included age, the APACHE score (failed weaning model only), duration of mechanical ventilation, RSBI, mean DE, and mean PIC thickening fraction. MedCalc version 14 and SPSS (version 26) for Microsoft Windows (Armonk, NY: IBM Corp.) were used for statistical analysis.

## Results

**Fig. 2:**
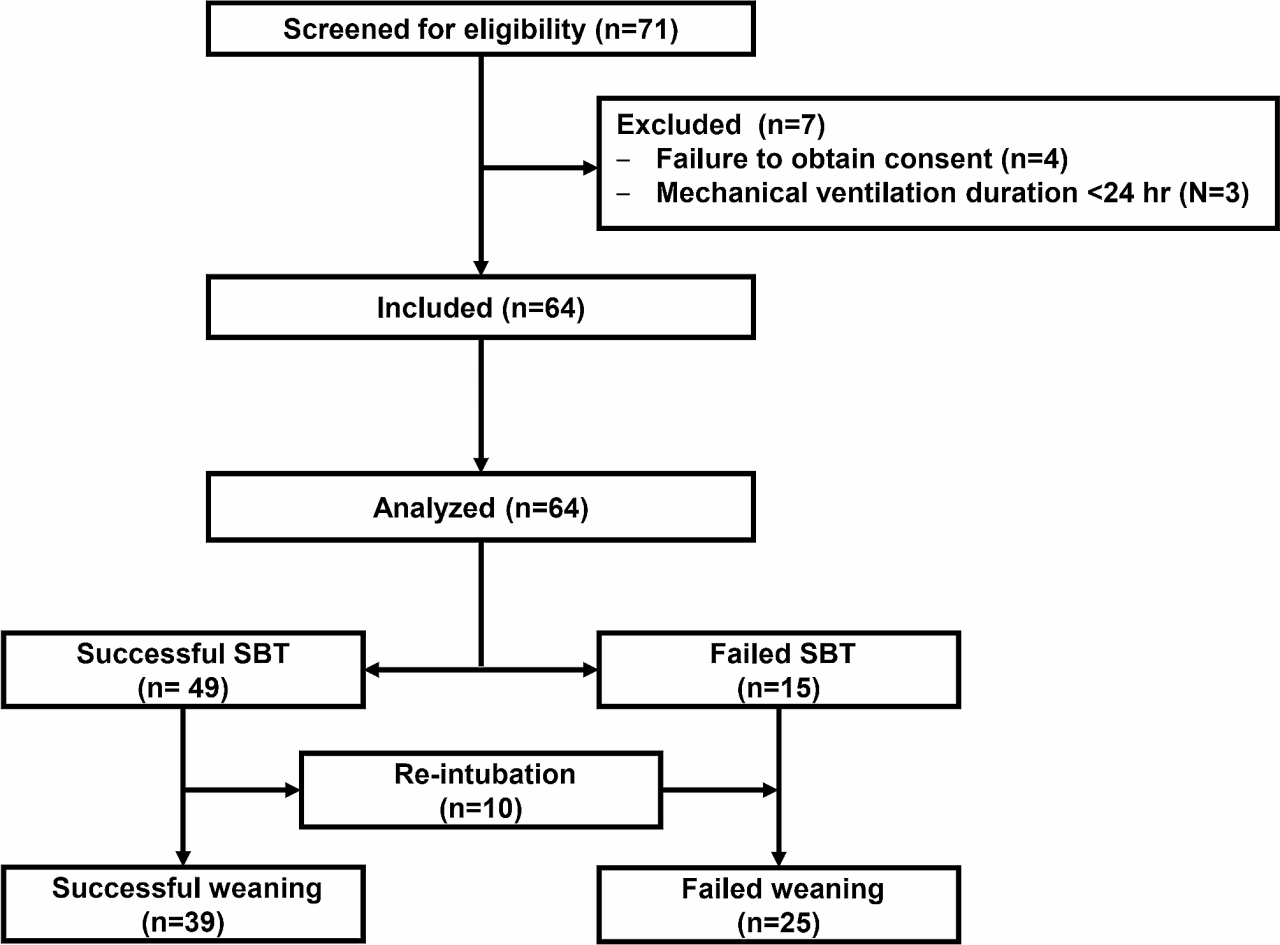
Patients’ enrollment flowchart

Among the 71 patients who fulfilled the weaning criteria, seven patients were excluded. Sixty-four patients were included and were available for final analysis; 15 patients had failed SBT. Forty-nine patients underwent successful SBT, and 10 of 49 (20%) patients were reintubated within 48 h (Fig. 2).

Patient demographic, clinical, and laboratory data are presented in Table 1. Hemodynamic instability and respiratory failure were the main causes of mechanical ventilation (Table 1).

**Table 1:** Demographic, clinical, ultrasound data and co-morbidities. Data are presented as mean (standard deviation), median (quartiles), and count (frequencies)

	All (*n* = 64)
Age (years)	51 (15)
Male gender	32 (50%)
Body mass index (Kg/m^2^)	28 (26–35)
Days of MV at inclusion	4 (3–8)
PaO_2_/FiO_2_	308 (240–363)
PaCO_2_ (mmHg)	39 (6)
Heart rate (beat/min)	83 (67–95)
Mean arterial pressure (mmHg)	70 (64–80)
Respiratory rate (breath/min)	22 (16–26)
RSBI	46 (28–69)
APACHE II	20 (8)
Co-morbidities	
Diabetes Mellites	27 (42%)
Hypertension	17 (26%)
Ischemic heart disease	12 (19%)
Hypothyroidism	3 (5%)
Chronic kidney disease	3 (5%)
Bronchial asthma	2 (3%)
Atrial fibrillation	2 (3%)
Initial Cause for mechanical ventilation	
Hemodynamic instability	50 (78%)
Respiratory failure	12 (19%)
Disturbed conscious level	2 (3%)
Source of sepsis	
Abdominal infections	27 (42%)
Soft tissue infection	25 (39%)
Respiratory	9 (14%)
others	3 (5%)
Ultrasound measurements	
Rt-DE (mm)	26 (12–31)
Lt-DE (mm)	22 (11–29)
M-DE (mm)	24 (11–29)
Rt-PIC thickening fraction (%)	5 (3–19)
Lt-PIC thickening fraction (%)	5 (3–18)
M-PIC thickening fraction (%)	6 (3–19)

APACHE II: Acute Physiology and Chronic Health Evaluation II,, Lt-DE: left diaphragmatic excursion, Lt-PIC: left parasternal intercostal muscle, M-DE: mean diaphragmatic excursion, M-PIC: mean parasternal intercostal thickening fraction, PaO_2_/FiO_2_: ratio of arterial oxygen partial pressure to fractional inspired oxygen, RR: Respiratory rate, RSBI: Rapid Shallow Breathing Index, Rt-DE: right diaphragmatic excursion, Rt-PIC: right parasternal intercostal muscle

The AUC (95% CI) for the ability of the right and left PIC thickening fraction to predict reintubation was 0.97 (0.88–1.00) and 0.96 (0.86–1.00), respectively, and for the prediction of failed weaning, the PIC thickening fraction AUC was 0.98 (0.91–1.00) and 0.97 (0.90–1.00), respectively (Table 2).

**Table 2:** The AUC analysis for the ability to predict failed weaning and reintubation

	AUC (95% CI)	Sensitivity % (95% CI)	Specificity% (95% CI)	PPV% (95% CI)	NPV% (95% CI)	Cut-off value
Re intubation (*n* = 10/49)						
Rt-PIC thickening fraction	0.97(0.88-1.00)	100(69–100)	92(79–98)	77(46–95)	100(90–100)	> 6.5%
Lt-PIC thickening fraction	0.96(0.86-1.00)	100(69–100)	92(79–98)	77(46–95)	100(90–100)	> 8.3%
Rt-DE	0.96(0.86-1.00)	90(56–100)	95(83–99)	82(48–98)	97(86–100)	≤ 18 mm
Lt-DE	0.95(0.85–0.99)	100(69–100)	77(61–89)	53(29–76)	100(88–100)	≤ 22 mm
Failed weaning (*n* = 25/64)						
Rt-PIC thickening fraction	0.98(0.91-1.00)	96(75–99)	97(87–100)	96(80–100)	97(86–100)	> 9.4%
Lt-PIC thickening fraction	0.97(0.90-1.00)	100(86–100)	92(79–98)	89(72–98)	100(90–100)	> 8.3%
Rt-DE	0.98(0.91-1.00)	96(80–100)	95(83–99)	92(75–100)	97(86–100)	≤ 18 mm
Lt-DE	0.98(0.91-1.00)	92(74–99)	95(83–99)	92(74–99)	95(83–99)	≤ 16

AUC: area under receiver operating characteristic curve, CI: confidence interval, DE: diaphragmatic excursion, Lt-PIC: left parasternal intercostal muscle, NPV: negative predictive value, PPV: positive predictive value, Rt- PIC: right parasternal intercostal muscle

At a cutoff value of > 6.5–8.3%, the PIC thickening fraction had a negative predictive value of 100% for the prediction of reintubation. The AUCs for the ability of the PIC thickening fraction to predict reintubation and failed weaning were comparable with those for DE (Table 2).

**Fig. 3:**
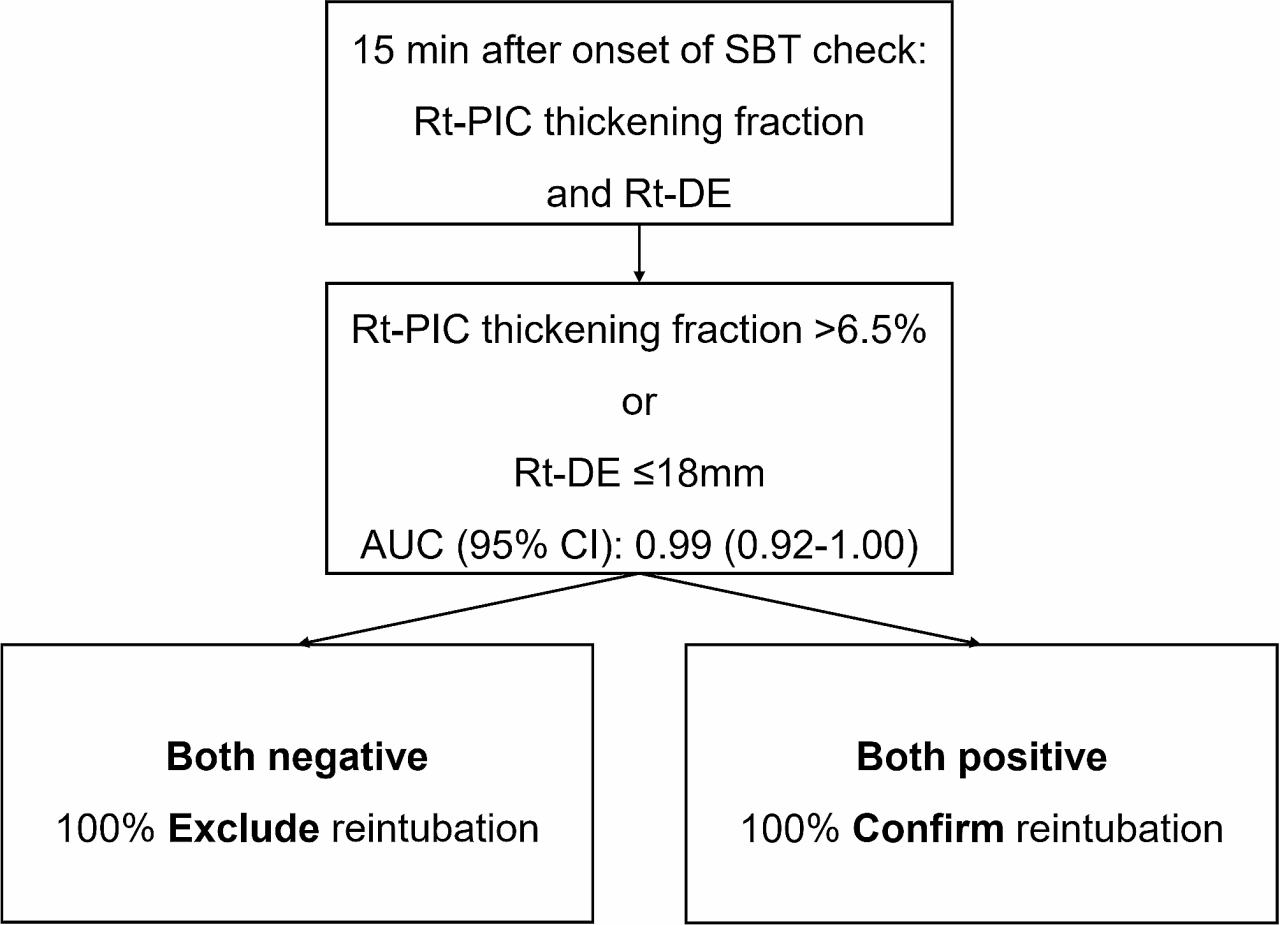
A proposed approach for utilizing the ultrasound assessment of both Rt-PIC thickening fraction and Rt-DE during the SBT in predicting reintubation within 48 h. AUC: area under receiver operating characteristic curve, CI: confidence interval, Rt-DE: right diaphragmatic excursion, Rt-PIC: right parasternal intercostal muscle, SBT: spontaneous breathing trial

The AUC (95% CI) of the model, including right PIC thickening fraction > 6.5% and right DE ≤ 18 mm, for predicting reintubation was 0.99 (0.92–1.00). The presence of one positive sonographic finding had a sensitivity of 100 (69–100)%, specificity of 87 (73–96)%, positive predictive value of 67 (38–88)%, and negative predictive value of 100 (90–100)%. The presence of both positive sonographic findings had a sensitivity of 90 (56–100)%, specificity of 100 (91–100)%, positive predictive value of 100 (66–100)%, and negative predictive value of 98 (87–100)%. Figure 3 presents the proposed algorithm for identifying the risk of reintubation based on right-side sonographic findings.

Univariate analysis revealed that increased age, duration of mechanical ventilation, respiratory rate, RSBI, PIC thickening fraction, and lower DE were risk factors for reintubation (Supplementary Tables 1 and 2).

Multivariate analysis, including the significant parameters from the univariate analysis, revealed that the independent predictors of reintubation and failed weaning were high PIC thickening fraction and low DE (Table 3).

**Table 3:** Multivariate analysis for failed weaning and reintubation

	Failed weaning (*n* = 25/64)		Reintubation (*n* = 10/49)	
	Odd ratio (95% CI)	*P*-value	Odd ratio (95% CI)	*P*-value
M-DE (mm)	0.59 (0.37–0.95)	0.028	0.60 (0.37–0.95)	0.031
M-PIC thickening fraction (%)	1.35 (1.02–1.78)	0.039	1.34 (1.01–1.78)	0.042

M-DE: mean diaphragmatic excursion, M-PIC: mean parasternal intercostal muscle

## Discussion

Our findings revealed that the PIC thickening fraction could accurately predict both failed weaning and reintubation in patients with sepsis. Furthermore, PIC was an independent risk factor for reintubation. We also found that a combination of impaired diaphragmatic function and increased PIC muscle activity could perfectly predict reintubation, whereas the absence of both findings could predict successful extubation. The role of SBT is to evaluate extubation readiness by examining the balance between ventilatory workload and drive. The intercostal muscles are accessory respiratory muscles that exhibit increased activity proportionate to excessive work of breathing [7]. This increased activity compensates for the impaired diaphragmatic function [7] leading to an inverse relationship between the PIC thickening fraction and DE [7, 17]. Thus, the PIC thickening fraction can be used as a tool for evaluating current or pending respiratory distress, with the advantage of being easier than diaphragmatic ultrasound indices. Supporting this explanation, Formenti et al. demonstrated that the PIC muscles contribute to the respiratory drive in patients with respiratory distress by impeding the paradoxical inward displacement of the ribs. Formenti et al. concluded that the presence of unphysiological activity of PIC muscles denotes excessive recruitment of accessory inspiratory muscles, which reflects respiratory distress [18]. In another study, the same authors found a significant decrease in the thickness of both the PIC muscles and diaphragm in the early stage of intensive care unit stay [19].

Previous data on the use of the PIC thickening fraction support our findings. Dres et al. found that PIC thickening could predict SBT failure [7], even in patients with normal diaphragmatic thickness [9]. PIC thickening also predicts noninvasive ventilation failure in patients with COVID-19 [20]. More recently, PIC thickening was found to be a good tool for risk stratification in the early phase of hospital admission in patients with COVID-19 [8]. This study provides new insights into the use of intercostal muscle ultrasound for being the first study to evaluate the ability of PIC thickening to predict reintubation. Reintubation is associated with critical airway and respiratory complications, making its prediction more crucial than a failed SBT [21–23]. Moreover, our study strictly included patients with sepsis who are at high risk of preferential respiratory muscle dysfunction due to reduced blood supply as well as direct damage to contractile proteins [12, 24].

Interestingly, the cutoff value in our study for the PIC thickening fraction (≈ 8%) was close to that of previous studies that evaluated the same measurements in other populations. Dres et al. reported that a PIC thickening fraction of > 9.5% during SBT could predict failure [7]. Helmy et al. found that a PIC thickening fraction of > 9% predicted noninvasive ventilation failure in patients with COVID-19 [20]; the same authors found that a PIC thickening of > 8.3% on hospital admission predicted the need for ventilatory support in patients with COVID-19 [8].

In this study, we assessed the ability to combine the PIC thickening fraction and DE to predict reintubation. We report that if none of the parameters were positive for reintubation, the occurrence of reintubation with 100% accuracy will be excluded, whereas if both parameters were positive for reintubation, the occurrence of reintubation with 100% accuracy will be confirmed. Furthermore, this model demonstrated excellent accuracy with a near-perfect AUC (AUC = 0.99). The cutoff values were obtained from the right-side intercostal muscle and hemidiaphragm. We selected the assessment of the right side for the proposed algorithm for several reasons. First, assessment of the right hemidiaphragm is easier than that of the left hemidiaphragm because of the presence of the liver, which provides a wider acoustic window than the spleen on the left side. Second, according to a recent expert consensus, unilateral assessment of the right hemidiaphragm is acceptable and can replace assessment of both sides [25]. Incorporating several variables into the assessment of respiratory status usually aims to achieve higher accuracy than the use of single variables [26]. Several combinations and ratios are commonly used in daily practice, such as the PaO_2_/FiO_2_ ratio and RSBI. More recently, the incorporation of respiratory muscle ultrasound-derived variables into classic parameters showed good results, such as the diaphragmatic RSBI [27]. According to our results, we found that the combination of DE and PIC can provide additional insights into respiratory evaluation during weaning.

Reintubation is a major adverse event that is independently responsible for poor outcomes in critically ill patients [22, 23]. Extubation failure is associated with a 5-fold increase in mortality [28]. Thus, predicting patients who are likely to fail is a hot topic in daily critical care practice. Early detection of high-risk patients could prevent premature extubation and enhance investigative and therapeutic measures for the cause of failure. High-risk patients for reintubation might also benefit from receiving noninvasive respiratory support after extubation. The use of noninvasive positive pressure ventilation and/or high-flow nasal oxygen reduced the incidence of failure after extubation [29–31]. However, these interventions are not devoid of disadvantages, such as cost, patient discomfort, and gastric insufflation; therefore, the use of noninvasive ventilation after weaning is still not routinely implemented in all patients, and it is unclear how to select patients who can benefit from it [32, 33]. High-risk patients for reintubation might be an appropriate subgroup that can benefit from prophylactic respiratory support after extubation to prevent reintubation [32, 33]. Our findings could help to identify these patients. The COVID-19 pandemic produced an unusual situation with overwhelming need for ventilators and intensive care beds. This crisis highlighted the importance of a rapid and efficient weaning process that provides appropriate extubation decision and avoid unnecessary delay in bed clearance for new admissions.

The use of point-of-care ultrasound is expanding exponentially in critical care medicine [34] because it is feasible at the bedside, noninvasive, and effective in the examination of several organs in a short period. Point-of-care ultrasound has become a basic skill for acute care physicians [35, 36]. The most common and easy ultrasound tool for measuring respiratory function is DE. Our results support the available literature regarding the accuracy of DE in predicting weaning failure and adding PIC thickening fraction, which showed nearly the same predictive values as DE with the advantage of being much easier. Furthermore, we introduced a novel combination of both DE and PIC to rule in and out successful extubation.

Our study has limitations, such as being a single-center observational study confined to a specific population with surgical sepsis. A single operator performed all ultrasound measurements; however, previous studies have shown that ultrasound assessment of respiratory muscle has good reproducibility [7]. Furthermore, we recorded the respiratory data and ultrasound measurements at a single time point during the SBT; therefore, temporal changes could not be assessed. Future larger studies are needed to confirm our findings in other types of patients and to assess ultrasound measurements at multiple time points during the SBT to identify the optimum timing of measurement and definitive cutoff value for implementation in clinical practice. We did not measure the maximum inspiratory pressure during the SBT; therefore, future studies are needed to assess the relationship between respiratory muscle function and maximum inspiratory pressure and the risk of reintubation.

In conclusion, among surgical patients with sepsis, PIC thickening fraction evaluated during the SBT is an independent risk factor for both weaning failure and reintubation. The PIC thickening fraction has an excellent predictive value for reintubation. A PIC thickening fraction of ≤ 6.5–8.3% can exclude reintubation with 100% accuracy. The combination of high PIC and low DE can also indicate a high risk of reintubation. However, larger studies that include different populations are needed to replicate our findings and validate the cutoff values.

### Electronic supplementary material

Below is the link to the electronic supplementary material.

Supplementary Material 1

## Data Availability

The datasets used and/or analyzed during the current study are available from the corresponding author on reasonable request.

## Abbreviations

APACHE: Acute Physiology and Chronic Health Evaluation
AUC: Area under receiver operating characteristic curve
BMI: Body mass index
CI: Confidence interval
COVID-19: Coronavirus disease
DE: Diaphragmatic excursion
MAP: Mean arterial blood pressure, M-DE: mean diaphragmatic excursion
M-PIC: Mean parasternal intercostal
MV: Mechanical ventilation NPV: negative predictive value
PPV: Positive predictive value
PIC: Parasternal intercostal muscle
RR: Respiratory rate
RSBI: Rapid shallow breathing index
SBT: Spontaneous breathing trial
SPSS: Statistical package for social science
