# Supplementary material for: Ability of parasternal intercostal muscle thickening fraction to predict reintubation in surgical patients with sepsis
Source: BMC Anesthesiol. 2024 Aug 22;24:294. doi: 10.1186/s12871-024-02666-8 (PMC11340156; doi:10.1186/s12871-024-02666-8)
Supplement: Supplementary file 1 — Supplementary Material 1 [file 12871_2024_2666_MOESM1_ESM.docx]

**Ability of parasternal intercostal muscle thickening fraction to predict reintubation in surgical patients with sepsis**.

Mina A Helmy (M.D.) ^a^, Ahmed M Hasanin (M.D, D.E.S.A.) ^a^, Lydia M Milad (MSc)^a^, Maha Mostafa (M.D.) ^a^, Walid I Hamimy (M.D.) ^a^, Rimon S Muhareb (M.D.) ^a^, Heba Raafat (M.D.) ^a^,

Supplementary table 1: Univariate analysis for reintubation. Descriptive data are presented as mean (standard deviation), median (quartiles), and frequency (%).

|  | Successful weaning (n=39/49) | Reintubation (n=10/49) | Odd ratio (95% CI) | *P*-value |
| --- | --- | --- | --- | --- |
| Age (years) | 46 (14) | 57 (12) | 1.06(1.01-1.12) | 0.029 |
| Male sex | 21 (43%) | 4 (8%) | 0.57(0.14-2.35) | 0.438 |
| BMI (kg/m^2^) | 28 (26, 34) | 29 (25, 37) | 1.01(0.91-1.13) | 0.989 |
| APACHE II | 17 (7) | 20 (7) | 1.07(1.00-1.19) | 0.187 |
| Days of MV | 3 (3, 5) | 8 (5, 9) | 1.64(1.20-2.24) | 0.002 |
| PaO_2_/FiO_2_ | 308 (240, 365) | 241 (225, 314) | 0.99(0.98-1.00) | 0.116 |
| PaCO_2_(mmHg) | 38 (6) | 41 (7) | 1.08(0.95-1.22) | 0.238 |
| Heart rate (beat/min) | 83 (66, 89) | 83 (78, 87) | 1.02(0.96-1.08) | 0.550 |
| MAP (mmHg) | 73 (65, 82) | 71 (61, 79) | 0.98(0.93-1.05) | 0.590 |
| RR (breath/min) | 17 (16, 22) | 26 (22, 28) | 1.32(1.10-1.60) | 0.004 |
| RSBI | 32 (24, 46) | 55 (44, 73) | 1.05(1.01-1.08) | 0.015 |
| M-DE (mm) | 28.0 (24.5, 33.5) | 15.0 (9.5, 17.1) | 0.64(0.46-0.88) | 0.006 |
| M-PIC thickening fraction (%) | 3.0 (2.5, 4.7) | 15.1 (11.4, 22.5) | 1.32(1.11-1.56) | 0.002 |

APACHE II: Acute Physiology and Chronic Health Evaluation II, BMI: body mass index, MAP: mean arterial blood pressure, M-DE: mean diaphragmatic excursion, M-PIC: mean parasternal intercostal muscle, MV: mechanical ventilation, PaO_2_/FiO_2_: ratio of arterial oxygen partial pressure to fractional inspired oxygen, RR: Respiratory rate, RSBI: Rapid Shallow Breathing Index.

Supplementary table 2: Univariate analysis for failed weaning. Descriptive data are presented as mean (standard deviation), median (quartiles), and frequency (%).

|  | Successful weaning (n=39/64) | Failed weaning (n=25/64) | Odd ratio (95% CI) | *P*-value |
| --- | --- | --- | --- | --- |
| Age (years) | 46 (14) | 58 (14) | 1.06(1.06-1.11) | 0.003 |
| Male sex | 21 (33%) | 11 (17%) | 0.67(0.25-1.85) | 0.443 |
| BMI (kg/m^2^) | 28 (26, 34) | 29 (25, 37) | 1.03(0.96-1.11) | 0.434 |
| APACHE II | 17 (7) | 24 (8) | 1.14(1.05-1.23) | 0.002 |
| Days of MV | 3 (3, 5) | 7 (5, 10) | 1.70(1.30-2.22) | <0.001 |
| PaO_2_/FiO_2_ | 308 (240, 365) | 308 (236, 363) | 1.00(0.99-1.00) | 0.465 |
| PaCO_2_(mmHg) | 38 (6) | 40 (6) | 1.06(0.97-1.16) | 0.223 |
| Heart rate (beat/min) | 83 (66, 89) | 83 (74, 91) | 1.02(0.98-1.06) | 0.401 |
| MAP (mmHg) | 73 (65, 82) | 70 (63, 75) | 0.97(0.93-1.02) | 0.247 |
| RR (breath/min) | 17 (16, 22) | 26 (24, 28) | 1.42(1.21-1.68) | <0.001 |
| RSBI | 32 (24, 46) | 69 (56, 84) | 1.07(1.04-1.11) | <0.001 |
| M-DE (mm) | 28.0 (24.5, 33.5) | 9.7(7.0, 14.3) | 0.60(0.44-0.82) | <0.001 |
| M-PIC thickening fraction (%) | 3.0 (2.5, 4.7) | 21.7 (15.4, 33.3) | 1.40(1.18-1.65) | <0.001 |

APACHE II: Acute Physiology and Chronic Health Evaluation II, BMI: body mass index, MAP: mean arterial blood pressure, M-DE: mean diaphragmatic excursion, M-PIC: mean parasternal intercostal muscle, MV: mechanical ventilation, PaO_2_/FiO_2_: ratio of arterial oxygen partial pressure to fractional inspired oxygen, RR: Respiratory rate, RSBI: Rapid Shallow Breathing Index.
